# Evaluation of the Participation of Community Pharmacists in Primary Healthcare Services in Nigeria: A Mixed-Method Survey

**DOI:** 10.34172/ijhpm.2020.224

**Published:** 2020-11-25

**Authors:** Maduabuchi R. Ihekoronye, Kanayo P. Osemene

**Affiliations:** Department of Clinical Pharmacy and Pharmacy Administration, Obafemi Awolowo University, Ile-Ife, Nigeria.

**Keywords:** Community Pharmacy Practice, Primary Healthcare, Service Quality, Pharmacy Service Management, Nigeria

## Abstract

**Background:** Achieving universal health coverage in poorly-resourced settings like Nigeria demands optimal mobilization of all healthcare resources including community pharmacists. Such efforts are hampered by insufficient data on primary healthcare (PHC) contributions by community pharmacists. The study aimed to identify PHC services offered by community pharmacists; assess impact of technologies on PHC service quality; and evaluate factors influencing management of PHC services in Nigeria.

**Methods:** A descriptive cross-sectional survey of 321 community pharmacies and 642 clients was undertaken between April and August, 2019. Semi-structured pre-tested questionnaires were administered on randomly-selected community pharmacists and clients. Interviews were conducted with key informants. Data were summarized using frequency and percentages while weighted averages on 5-point ordinal scales and chi-square tests were used to identify weights and associations between variables respectively at *P*<.050.

**Results: **Response rates of pharmacists and clients were 74.7% (N=321) and 100% (N=642); while their median ages were 39.41 and 51.20 years respectively. Community pharmacists offered services in all eight domains of PHC, especially supply of medicines for treating of endemic diseases (mean weighted average [MWA]=4.59), and disease prevention (4.54) but least of vaccine administration (2.39). Blood glucose screening devices were the most adopted technology with significant impact on service quality (χ^2^ 6.86, *P*=.030). Major challenges to management of PHC services were poor awareness of pharmacists’ roles (4.31) and lack of integration with the PHC infrastructure (4.31). Capacity constraints in finances (4.11), technologies (4.09), and human resources (3.99) were significant. However, major facilitators were pharmacists’ managerial skills (4.35), and strong client relationships (4.27).

**Conclusion:** In Nigeria, community pharmacists offered important PHC services. Deploying technologies were associated with improved service quality. If community pharmacists are integrated in the national PHC architecture and financial incentives are provided, their competences and goodwill would enhance the achievement of universal health coverage.

## Background

**Key Messages**

**Implications for policy makers**

- Community pharmacists are underutilized primary healthcare (PHC) service providers.
- Their concentration in urban centres remains a significant challenge for universal health coverage.
- Policy action is required to incentivize establishment of community pharmacies in rural communities.
- Facilitating community pharmacists’ access to relevant digital technologies will significantly improve their contribution to PHC.
- Integrating community pharmacists in the PHC architecture will significantly improve access to care and overall health outcomes in Nigeria.

**Implications for the public**

Community pharmacies are private-sector facilities providing spatial proximity and easy accessibility to quality primary healthcare (PHC) services to people in their communities. With inadequate capacities in public sector facilities, people should have an option to access certain PHC services in their community pharmacies. If community pharmacies are integrated with the public sector service providers, patients will enjoy improved continuity of care with easy referrals along both vertical and horizontal channels of care.

Community pharmacies are private sector healthcare facilities, under the direct oversight of registered pharmacists, and offer a wide range of primary healthcare (PHC) services in addition to the traditional dispensing of prescribed medications.^1^ Community pharmacists are the most accessible healthcare professionals located in the very hearts of communities close to where people live, work and play.^2^ Since spatial proximity has been shown to enhance access to healthcare services and overall health outcomes, it may be argued that maximizing the PHC contributions of community pharmacists will significantly enhance the attainment of universal health coverage.^3,4^ In Nigeria (a low- and middle-income country), about 95% of community pharmacies are independent, bricks-and-mortar, retail outlets with the size of small and medium scale enterprises.^5^ Most of them operate as “silos” in their communities, not connected with the PHC architecture of the country.^6^ Hence efforts at achieving universal health coverage are hampered not only by shortages in human resources for health and access points for PHC but also by dearth of evidence on the PHC contributions by community pharmacists.^7^

 From the Alma Ata Declaration of 1978 to date, PHC have proven to be an important framework for improving access to quality healthcare services, particularly in the developing countries. PHC encompasses a comprehensive blend of promotive, preventive, protective, curative, rehabilitative, and palliative care made available throughout the life course, addressing the broader determinants of health (social, economic, environmental, and people’s characteristics and behavior), using evidence-based public policies while empowering individuals, families and communities to play active roles at every stage.^8^ There are essentially eight domains of PHC including health education (directed at prevailing health problems and methods to identify, prevent and control them); prevention and control of locally endemic diseases; treatment of communicable and non-communicable diseases, including mental health; supply of essential medicines; immunization against major infectious diseases; maternal and child health, including family planning; provision of clean water and sanitation; and provision of safe and basic nutrition and nutritional supplements.^8^

 If properly deployed, digital technologies have the capacity to facilitate access to PHC services to underserved communities.^9^ Beyond improved access, there is also the issue of improved quality of PHC services with due attention given to safety, effectiveness, people-centredness, equity, timeliness, and efficiency. There is an emerging consensus that attaining these goals may be impossible without the adoption of appropriate digital technologies including point-of-care diagnostics.^9,10^ In Nigeria, community pharmacists usually attend to the sick, the well and apparently well in the communities making them suitable agents for health education, disease prevention, and proactive case finding. While digital technologies have been shown to enhance these services, ^9^there is limited data on their level of adoption by community pharmacists.There is also the need to examine to what extent these technologies impact on quality of PHC services offered by the community pharmacists.

 While several theoretical frameworks exist in literature in relation to the adoption of relevant technologies, the Technology Acceptance Model^11^ seems most appropriate for the community pharmacy scenario because of its ability to calibrate the fit between technology and user tasks, and the power to predict use, individual intention to perform a behavior and accept technology. According to Technology Acceptance Model, subject to certain variables in the external environment, the attitude of community pharmacists towards a new technology will depend on its perceived usefulness and ease of use (Figure). A positive attitude will reflect in a favorable behavioral intention to use, and actual deployment of the technology. The modifications to the original model, relevant to community pharmacy include differentiating the external variables into social influence, (subjective norm, voluntariness, and image), cognitive instrumental processes (job relevance, output quality, and result demonstrability), and experience.^12,13^

**Figure:**
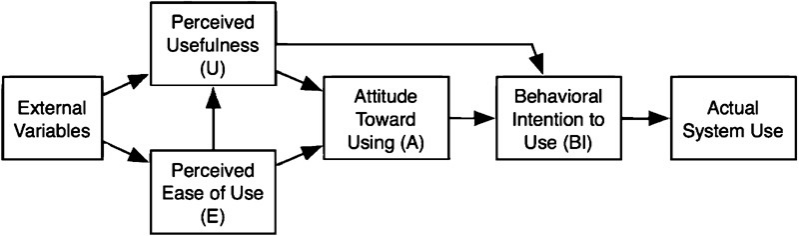
Technology Acceptance Model. Reprinted with permission from Davies.^11^

 Effectiveness of PHC interventions by community pharmacists depend to a large extent on contextual variables within and outside the practice environment. These factors, though poorly understood at the moment, are important determinants of the acceptability of the PHC services by clients in local practice settings.^14,15^It is therefore necessary to understand the relevant factors that must be taken into account in the planning, implementation and evaluation of the PHC contributions of community pharmacists in Nigeria.

 The study identified PHC services offered by community pharmacists, assessed the impact of technologies on service quality and evaluated the factors affecting the management of these services in Nigeria.

 This study is significant as it provides evidence to guide policy-makers and regulators in their efforts to maximize efficiencies in the health sector and improve access to quality PHC services in the era of universal health coverage. Community pharmacists will also find guidance in managing contextual factors affecting the quality of services they render to their clients.

## Methods

###  Study Design

 Mixed-method design was employed. A questionnaire-guided cross-sectional survey of community pharmacies was carried out between April and August 2019. Three hundred twenty-one randomly-selected community pharmacists were surveyed alongside 642 clients who were selected by accidental sampling method on the basis of two clients per pharmacy. Three community pharmacists were purposively- selected as key informants for oral interviews.

###  Study Setting

 The study was focused on community pharmacies which represent the most prominent specialty of pharmacy practice employing about 59.8% of all pharmacists in Nigeria. Data from the Pharmacists Council of Nigeria were used to determine size of study participants.^16^

###  Study Area

 The study area was the Southwestern geopolitical zone of Nigeria comprising the 6 States of Lagos, Ogun, Oyo, Osun, Ondo and Ekiti with a total population of about 32 million.^17^ The zone was chosen partly because of feasibility of data collection and partly because it is home to about 50% of all community pharmacies in Nigeria and over 60% of the drug manufacturing and importation outfits from where community pharmacists source their products.^18^

###  Inclusion Criteria

 Only retail pharmacies were included. Pharmacists whose first date of registration as community pharmacists was 2018 or earlier were included. This was to ensure they have had at least one full year practice experience in providing PHC services. Moreover, only those community pharmacist respondents with a corresponding set of two client respondents available were included.

###  Exclusion Criteria

 Pharmaceutical wholesalers were excluded from the study since they do not interact directly with patients. Community pharmacists with less than one full year of practice experience were excluded. Pharmacists in other practice settings and other geopolitical zones (outside the Southwest) were excluded, even if they offered PHC services.

###  Sample Size Determination

 The sample size was calculated using the Cochran (1977) formula assuming a 5% error rate.^19^ Sample size from each State was determined in line with the proportion of the State relative to the total population of 1941 from the entire Southwest in the register of Pharmacists Council of Nigeria as at December 31, 2018.

 The Cochran formula:


$$n_{0}=\frac{Z^{2}pq}{e^{2}}$$

Where n_0_ = Cochran’s estimated sample size; e = margin of error (which was 0.05); p = confidence level (95% or 0.5); q = 1-p(0.5); Z = value in the Z table (which was 1.96); no = (1.96)^2^(0.5)(0.5)/(0.05)^2^=385

 For a finite population of 1941, we use the modified Cochran’s formula:


$$n=\frac{n_{0}}{1+\frac{\left(n_{0}-1\right)}{N}}$$

Where n = required sample size; no = estimated sample size from above (385); N = population (1941); n = 385/ 1 + (384/1941) = 320

###  Sampling Techniques

 Simple random sampling technique was employed to select the community pharmacists. Two clients of each pharmacy were selected by accidental sampling based on whoever happened to visit the pharmacy at the time data collectors were around, provided such clients gave their informed consent. The three key informants were purposively selected in line with the study criteria and based on recommendations from the Association of Community Pharmacists of Nigeria.

###  Questionnaire Design

 Two sets of questionnaires were deployed to collect primary data, one for the community pharmacists and the other for their clients. An interview guide was used for the oral interviews. The questionnaire for community pharmacists was adapted from extensive review of previous studies.^17-19^It sought information about demographic characteristics of respondents, the PHC services offered, the technologies deployed to provide these services, and what factors the pharmacists considered as hindrances and facilitators to the management of the PHC services. Five-point Likert scales were used to identify important PHC services and factors affecting their management. For the PHC services, the 5-point Likert scale represents “Never”- never offered the service; “Rarely”- offers the service at least once in several months; “Sometimes”- offers the service at least once every month; “Often”- offers the service at least once every week; and “Always”- offers the service at least once daily. For factors affecting management of PHC services, the 5-point Likert scale represents “Strongly agree”- agree in every way; “Agree”- agree to some extent; “Can’t say”- not sure; “Disagree” disagree to some extent; Strongly disagree”- disagree in every way.

 The oral interviews were directed at key informants who were recommended by their State Chairmen of the Association of Community Pharmacists of Nigeria for their considerable experience and expertise in the provision of PHC services in their pharmacies. Their evidence was meant to strengthen data obtained from the questionnaires. In terms of service quality, the clients of community pharmacies were surveyed using an adapted standard (SERVQUAL) instrument,^23^ which was named Customer Assessment of Service Quality (CASQUAL). The CASQUAL instrument was translated to the local language (Yoruba), commonly spoken in all 6 States of interest for the benefit of those clients who could not communicate effectively in English.

###  Validation of Research Instruments

 Validity of research instruments was ensured by the expert scrutiny of senior faculty members whose comments and corrections were used to improve the quality of the questionnaires, particularly in the arrangement of the sections and the local language translation. Reliability of the questionnaires was determined using a pilot survey of 40 community pharmacists (alongside 2 clients each) located outside the study area. The sample size of 40 was chosen being up to the required 10% of sample size for the main study.^24^ Data from the pilot study was used to compute the reliability coefficient of the instruments. The Cronbach’s alpha coefficients showed internal consistency above the minimum cut off ≥0.7 for all the sections of the questionnaire. ^24,25^

###  Data Collection

 Research assistants were recruited and trained to collect data. An important precondition for recruitment was proficiency in both written and oral English and the local (Yoruba) languages. The researchers personally interviewed the key informants using an interview guide and the dual-moderator technique. One moderator kept the interviews flowing smoothly while the other ensured all the sections were covered. All the interviews were tape-recorded and transcribed verbatim for content analysis. Trained research assistants moved into the field to administer questionnaires on selected community pharmacists and their clients at the same time period. All respondents gave their informed consents in writing. Key informants chose the dates and times for their interviews and agreed on durations. Where duration was extended, it was with their consent.

###  Data Analysis

 Data obtained from the instruments was coded, sorted, and carefully cross-checked for data management using Excel spreadsheet. It was then exported to the Statistical Package for Social Sciences (SPSS) version 25 for Windows software for analysis at *P*< .050. Demographic characteristics of respondents were presented as frequency, percentages, and median scores. Weighted averages ± SD (standard deviation) and rankings were used to analyze the PHC services. Pairwise *t* test was used to compare expected and actual service quality. Level of adoption of technologies, as well as factors influencing PHC service management, were presented as frequencies and percentages. Chi-square test was used to establish association between variables. Content analysis was conducted on qualitative data from key informants. Important terms, phrases and concepts were coded in terms of frequency and patterns. The principal investigator and another senior faculty member validated the coding and the conclusions drawn from them.

## Results

 Demographic data of respondents is presented in Table 1. Out of a total of 430 community pharmacists who received the questionnaires, 321 (74.7%) responses were correctly completed and included in the study. Of these, 210 (65%) were males while 111 (35%) were females. For every pharmacist response included, there were 2 corresponding clients’ responses (questionnaires) correctly completed, making 642 client responses of which 605 (94.2%) were in English and 37 (5.8%) were in Yoruba language. Pharmacists and clients spent an average of 15 and 10 minutes respectively in filling the questionnaires. Key informants (2 females and 1 male) had 100% response rate while the mean duration of the interviews was 43 minutes (range 38-52 minutes). Median ages for pharmacists and clients were 39.41 and 51.20 years respectively. Educational qualification of the pharmacists was broadly categorized as either basic or specialized. Those with only the Bachelor of Pharmacy degree or its equivalent (B.Sc. Pharmacy) were considered basic, while those with additional higher degrees were categorized as specialists. About three quarters 235 (73%) had basic pharmacy education while one quarter 86 (27%) were specialists. The largest group 198 (62%) of the pharmacists were young professionals in their first decade of practice experience.

**Table 1:** Demographic Data of Community Pharmacist Respondents (N = 321)

**Variables**	**No.**	** %**
Gender		
Male	210	65.4
Female	111	34.6
Level of education		
B.Pharm./B.Sc.(Pharm.) only	235	73.2
M.B.A./M.Sc. and other higher degrees	86	26.8
Age (y)		
40 and below	178	55.4
Above 40	143	44.6
Years of community pharmacy practice experience		
1-10	198	61.7
11-20	89	27.7
Above 20	34	10.6
Location of practice		
Rural	57	17.8
Urban	264	82.2
Average daily customer count		
1-50	86	26.8
51-100	149	46.4
Above 100	86	26.8
Type of community pharmacy		
Wholesale	36	11.2
Independent retail	235	73.2
Wholesale plus retail	29	9.0
Retail chain	21	6.5

 As shown in Table 2, the 30 different PHC services of interest were categorized according to their corresponding domains in the universe of PHC. It will be noticed that some individual service items had aspects of more than one domain of PHC. The services were ranked by their mean scores into three groups of 10 corresponding to highly, moderately and least offered services. While malaria treatment was the most common PHC service offered, vaccine administration was the least.

**Table 2:** PHC Services Offered by Community Pharmacists (N = 321)

**PHC Service**	**Mean**	**SD**	**Rank**	**Domain of PHC**
Treating malaria with ACTs	4.59	0.71	1	Treatment/supply of drugs
Blood pressure checks	4.54	0.78	2	Disease prevention
Patient counselling during every dispensing	4.43	0.70	3	Health education
Treatment of common cold or flu	4.32	0.78	4	Treatment
Participate in drug therapy for chronic non-communicable disease	4.26	0.69	5	Treatment
Blood glucose screening	4.23	0.94	6	Disease prevention
Diarrhoea management with ORS	4.21	0.74	7.5	Treatment/maternal and child health
Educate customers to wash hands frequently	4.21	0.89	7.5	Health education/disease prevention
Participate in drug therapy of infectious disease	4.18	0.80	9	Treatment
Referrals for antenatal care	4.14	0.90	10	Maternal and child health
Syndromic management of STDs	4.08	0.92	11.5	Treatment
Ordering routine laboratory test	4.08	0.99	11.5	Treatment
Education on clean water and sanitation	4.07	0.95	13	Health education/water and sanitation
Supply of essential medicines and dressings	4.05	0.98	14	Supply of drugs
Counsel customers to handle and prepare food safely	4.01	0.94	15.5	Health education/nutrition
Generic substitution	4.01	0.82	15.5	Supply of drugs
Advice on herbal medicines	3.99	1.04	17	Health education/supply of drugs
Clean and disinfect commonly used surfaces	3.90	0.97	18.5	Health education/prevention and control of disease
Screening for drug interactions	3.90	0.89	18.5	Supply of drugs/health education
Provision and counselling on insecticide-treated mosquito nets	3.84	1.00	20.5	Prevention and control/health education
Detection of ADRs	3.84	0.94	20.5	Supply of drugs/health education
Do not share personal items	3.79	1.06	22	Health education/prevention and control
Cough and sneeze into a tissue or sleeve	3.72	1.08	23	Health education/prevention and control
Dressing minor wounds	3.69	1.16	24	Treatment
Get vaccinated	3.66	1.09	25	Disease prevention/immunization
Use of family planning devices	3.63	1.03	26	Maternal and child health/health education
Body mass index check	3.49	1.13	27	Disease prevention/health education
Avoid touching wild animals	3.30	1.20	28	Health education/prevention and Control
Blood cholesterol screening	3.02	1.50	29	Disease prevention/health education
Vaccine administration	2.39	1.59	30	Immunization

Abbreviations: PHC, primary healthcare; ACTs, artemisinin combination therapies; SD, standard deviation; ORS, oral rehydration salt; STDs, sexually transmitted diseases; ADRs, adverse drug reactions.

 In order to assess the difference between the expected and actual service quality, a paired samples *t* test was carried out to assess the difference in mean scores of service quality as expressed by clients. Table 3 presents the result which shows a significant gap between customer expectations and service fulfilment (t = 14.59; *P*< .050).

**Table 3:** Difference Between Expected and Actual Service Quality as Perceived by Clients (N = 642)

**Service quality**	**Mean**	**SD**	**t**	***P*** ** Value**
Expected	54.79	5.23	14.59	.001^a^
Actual	47.07	8.75		

Abbreviation: SD, standard deviation. ^a^ Significant.

Table 4 has information from two different groups of respondents. While information on technologies for PHC was obtained from community pharmacists, data for service quality were extracted from clients’ responses. The study applied the service quality (SERVQUAL) model to assess customer perceptions of PHC service quality. The model measured the gap between the quality of service expected and that of the actual service received, using domains of reliability, assurance, tangibles, empathy, and responsiveness.^23^ The smaller the gap, the higher the service quality. Composite measures of quality were categorized as low, moderate or high, and the association of technologies with these service quality indicators was examined.

**Table 4:** Technologies for PHC Services and their Association With Service Quality (N = 321)

**Item**	**Responses, No. (%)**		**Association With Service Quality, No. (%)**			**χ** ^ 2 ^	***P*** ** Value**
	**No**	**Yes**	**Low (≤ 34.00)**	**Moderate (35.00-51.00)**	**High (52.00+)**		
Stethoscope	29 (9.03)	292 (90.97)	26 (8.90)	160 (54.79)	106 (36.30)	0.340^a^	.844
Sphygmomanometer	31 (9.66)	290 (90.34)	26 (8.97)	160 (55.17)	104 (35.86)	0.017^a^	.991
Thermometer	27 (8.41)	294 (91.59)	23 (7.82)	162 (55.10)	109 (37.07)	5.922^a^	.052
Blood glucose screening device	6 (1.87)	315 (98.13)	27 (8.57)	173 (54.92)	115 (36.51)	6.860^a^	.032^b^
Bathroom scale/weighing balance	15 (4.67)	306 (95.33)	27 (8.82)	170 (55.56)	109 (35.62)	0.572^a^	.751
Dressing kit/first aid box	27 (8.41)	294 (91.59)	26 (8.84)	159 (54.08)	109 (37.07)	2.543^a^	.280
Wear hand gloves during physical exam	30 (9.35)	291 (90.65)	28 (9.62)	155 (53.26)	108 (37.11)	4.979^a^	.083
Blood cholesterol screening device	153 (47.66)	168 (52.34)	12 (7.14)	88 (52.38)	68 (40.48)	4.010	.135
Website	203 (63.24)	118 (36.76)	12 (10.17)	63 (53.39)	43 (36.44)	0.390	.823
Social media handle	149 (46.42)	172 (53.58)	13 (7.56)	95 (55.23)	64 (37.21)	1.092	.579
Social media platform	103 (32.09)	218 (67.91)	18 (8.26)	120 (55.05)	80 (36.70)	0.600	.741
Bulk SMS	179 (55.76)	142 (44.24)	15 (10.56)	74 (52.11)	53 (37.32)	1.242	.537
Computerized operations	63 (19.63)	258 (80.37)	23 (8.91)	141 (54.65)	94 (36.43)	0.214	.899
Documentation of patient interactions	124 (38.63)	197 (61.37)	20 (10.15)	102 (51.78)	75 (38.07)	0.247	.291
Software for drug interactions	210 (65.42)	111 (34.58)	18 (16.22)	54 (48.65)	39 (35.14)	0.311	.721

Abbreviations: PHC, primary healthcare; SMS, short message service. ^a^ Likelihood ratio used; ^b^ Significant at.05 level of significance.

 Item performance and ranking of factors affecting the management of PHC services, as expressed by community pharmacists, are presented in Tables 5 and 6 respectively.

**Table 5:** Item Performance of Factors Affecting Management of PHC Services in Community Pharmacies (N=321)

**Factors**	**Responses, No. (%)**				
	**Strongly Disagree**	**Disagree**	**Can’t Say**	**Agree**	**Strongly Agree**
Lack of awareness of CP role by other PHC professionals	12 (3.7)	13 (4.0)	8 (2.5)	118 (36.8)	170 (53.0)
Lack of integration of CP with PHC system	8 (2.5)	7 (2.2)	15 (4.7)	140 (43.6)	151 (47.0)
Lack of effective team approach to patient care in Nigeria	12 (3.7)	9 (2.8)	15 (4.7)	135 (42.1)	150 (46.7)
Scarcity of skilled human resources	14 (4.4)	23 (7.2)	24 (7.5)	151 (47.0)	109 (34.0)
Lack of financial capacity to hire and retain skilled personnel	10 (3.1)	17 (5.3)	16 (5.0)	164 (51.1)	114 (35.5)
Inadequate floor space in the pharmacy	22 (6.9)	36 (11.2)	30 (9.3)	153 (47.7)	80 (24.9)
Insufficient time to render PHC services	10 (3.1)	45 (14.0)	27 (8.4)	145 (45.2)	94 (29.3)
Unsure of profitability of non-drug services	16 (5.0)	39 (12.1)	37 (11.5)	125 (38.9)	104 (32.4)
Unsure of sustainability of PHC services	18 (5.6)	30 (9.3)	26 (8.1)	166 (51.7)	81 (25.2)
Poor payment systems	10 (3.1)	22 (6.9)	31 (9.7)	148 (46.1)	110 (34.3)
Lack of access to patient medical records	12 (3.7)	16 (5.0)	28 (8.7)	168 (52.3)	97 (30.2)
Regulatory limitations to clinical services by CPs	18 (5.6)	21 (6.5)	40 (12.5)	146 (45.5)	96 (29.9)
Insufficient clinical education and training during undergraduate degree	13 (4.0)	38 (11.8)	28 (8.7)	127 (39.6)	115 (35.8)
Lack of adequate infrastructure and technologies for PHC	12 (3.7)	13 (4.0)	20 (6.2)	164 (51.1)	112 (34.9)
Poor patients'/public perception of CP as a PHC professional	10 (3.1)	22 (6.9)	25 (7.8)	143 (44.5)	121 (37.7)
CPs are not inhibited by bureaucracy in decision-making for PHC services	12 (3.7)	27 (8.4)	29 (9.0)	143 (44.5)	110 (34.3)
PHC services are not new to community pharmacists	6 (1.9)	15 (4.7)	17 (5.3)	145 (45.2)	138 (43.0)
CPs already have strong relationship with their clients	8 (2.5)	20 (6.2)	10 (3.1)	123 (38.3)	160 (49.8)
Customers trust their CPs	6 (1.9)	6 (1.9)	11 (3.4)	189 (58.9)	109 (34.0)
By their training, CPs have enough skills to manage PHC services	6 (1.9)	16 (5.0)	14 (4.4)	109 (34.0)	176 (54.8)

Abbreviations: PHC, primary healthcare; CP, Community pharmacist.

**Table 6:** Ranking of Factors Affecting Management of PHC Services in Community Pharmacy

**Factors**	**Mean**	**SD**	**Rank**
**Challenges**			
Lack of awareness of CP role by other PHC professionals	4.31	0.98	1.5
Lack of integration of CP with PHC system	4.31	0.86	1.5
Lack of effective team approach to patient care in Nigeria	4.25	0.95	3
Lack of financial capacity to hire and retain skilled personnel	4.11	0.94	4
Lack of adequate infrastructure and technologies for PHC	4.09	0.95	5
Poor patients'/public perception of CP as a PHC professional	4.07	1.00	6
Poor payment systems	4.02	1.00	7
Lack of access to patient medical records	4.00	0.96	8
Scarcity of skilled human resources	3.99	1.05	9
Insufficient clinical education and training during undergraduate degree	3.91	1.13	10
Regulatory limitations to clinical services by CPs	3.88	1.09	11
Insufficient time to render PHC services	3.83	1.09	12
Unsure of profitability of non-drug services	3.82	1.16	13.5
Unsure of sustainability of PHC services	3.82	1.09	13.5
Inadequate floor space in the pharmacy	3.73	1.16	15
**Facilitators**			
By their training, CPs have enough skills to manage PHC services	4.35	0.92	1
CPs already have strong relationship with their clients	4.27	0.97	2
PHC services are not new to community pharmacists	4.23	0.89	3
Customers trust their community oharmacists	4.21	0.76	4
CPs are not inhibited by bureaucracy in decision-making for PHC service	3.97	1.05	5

Abbreviations: PHC, primary healthcare; CP, Community pharmacist; SD, standard deviation.

###  Content Analysis of Key Informant Interviews

 Below are key statements by interviewees regarding PHC services and related technologies that give them visibility and uniqueness in their communities. They believe expanding these services would enhance public perception of community pharmacists as PHC service providers.

*“…Preventive health services give us the greatest mileage in our community pharmacy. We do a lot of disease parameter screening using technologies”* (Respondent from Ibadan, Oyo State).

*“So many people come to us for medicines and health related information, that’s how we demonstrate our expertise. Our regular health education services probably impact our community the most”* (Respondent from FESTAC Town, Lagos State).

*“Advocacy for vaccine uptake, cardiovascular risk assessment, disease prevention- screening for prostate cancer, breast cancer…these constitutes our major PHC services*” (Respondent from FESTAC Town, Lagos State).

*“…the test and treat campaign for malaria has become routine here. We also offer immunization services as well as screening for adverse drug reactions” *(Respondent from Akure, Ondo State).

*“…We have quite some specialty in providing information and commodities for family planning services including safe sex, child spacing, condom use, breastfeeding techniques…” *(Respondent from Akure, Ondo State).

###  Factors Affecting the Management of Primary Healthcare Services in Community Pharmacies

 Key informants unanimously identified the following factors:

- Retaining skilled workforce
- Infrastructural deficit in practice environment
- Poor societal perception of the pharmacist as a PHC service provider
- Poor payment systems
- Capacity limitations associated with ‘stand-alone’ practice
- Competency gap in providing and managing PHC services in-pharmacy
- Lack of systematic documentation of professional services including PHC services
- Lack of clarity on legal framework for vaccine administration by community pharmacists

###  Recommendations for Improving Primary Healthcare Service Quality in Community Pharmacies

 Most frequent and common recommendations by key informants include:

- Continuous training of community pharmacists
- Collaborative practice among community pharmacists
- Collaborative practice with Family Physicians
- Undergraduate pharmacy training to focus on entrepreneurship
- More political advocacy for inclusion of pharmacists in health sector governance
- Developing uniform framework for documentation of customer interactions
- Legislation to clearly empower community pharmacists to administer vaccines

## Discussion

###  Primary Healthcare Services in Nigeria Community Pharmacy

 In this study, through literature search and expert interviews, the services offered by community pharmacists were examined in the context of their fit within the eight established domains of PHC. The 30 PHC-related services identified were weighted in terms of frequency of occurrence. About one half of the highly-offered services (ranked 1-10) were found to be domiciled in treatment and control of endemic diseases, with malaria treatment as the singular most offered PHC service. This finding supports long held positions that effective control of malaria requires active participation of all community-based healthcare professionals.^26,27^ Disease preventive services (blood pressure check, among others) accounted for about 30% of the highly offered services; while 20% were related to health education. Maternal and child health and medicines supply were prominent. Patient counselling (ranked overall third) offers community pharmacists important opportunities for health education, cardiovascular risk assessment, screening for drug interactions and detection of adverse drug reactions, among others. What is often overlooked is the enormous potential for community pharmacists to leverage their easy accessibility to create even more value for these highly-offered PHC services, by collaborating with other healthcare providers in the public and public sector, acting as proactive case finding and referral hubs within their communities. The coronavirus disease 2019 (COVID-19) pandemic makes a strong case for this shift in practice paradigm.

 Of the moderately offered PHC services (ranked 11-20), 70% were related to health education; 50% to supply of essential medicines; 20% each to disease prevention and treatment of diseases respectively; while 10% each were related to nutrition and water/sanitation respectively. Drug-related aspects of PHC namely, supply of essential medicines and dressings, generic substitution and advise on herbal medicines, all belong to the moderately offered services. While it is easy to put a price on medicine items on the shelves of a community pharmacy, it is difficult to quantify the non-drug professional services offered by community pharmacists for the purpose of pricing.^28^ With predominant out-of-pocket mode of payments and low health insurance penetration, there is urgent need to evolve robust healthcare financing systems that pay appropriate fees for service to community pharmacists if they are to maintain the balance between professional and business practices required for improved outcomes.^29^That community pharmacists traditionally collect no consultation fees from their clients makes this fact even more significant.

 The least offered PHC services (ranked 21-30) were majorly related to disease prevention (60%) and health education (60%); immunization services (20%); treatment (10%) and maternal and child health (10%). It is important to emphasize that vaccine administration represents the least offered PHC service among the community pharmacists. This runs against obvious gains experienced in health outcomes engendered by the participation of community pharmacists in other climes such as the United States,^30^ United Kingdom,^31^ Australia,^32^ and South Africa.^33,34^

 The findings of the survey agree with the positions of key informants particularly in the under-explored potentials of community pharmacists for immunization services. The evidence equally underscore the claim that community pharmacy clients are getting more sophisticated such that a significant gap exists between customer expectations and service fulfilment (t = 14.59, *P* = .001). The data re-enforce earlier evidence.^3,20,31,32^In summary, it may be concluded that community pharmacists are PHC service providers, actively involved in all eight domains of PHC.

###  Adoption of Technology for Primary Healthcare Services in Community Pharmacy

 Blood glucose screening device was the most commonly deployed technology as almost all the respondents (315 or 98%) had and used them, with significant impact on service quality (6.68, *P* = .032). However, 153 (48%) of the community pharmacists had no cholesterol screening devices which represents a significant setback in their effort to provide quality primary care and empower their clients for self-care. It is significant to note that of the 52% that used these machines, only 7% were rated low in service quality while 40% were rated high and 52% moderate. Based on the modified technology acceptance model, the poor deployment of cholesterol screening devices could be due to unfavorable subjective norms and inadequate experience with digital technologies. Moreover, poor demand for screening services due to poor public perception of the community pharmacist, and lack of financial capacity to hire/retain skilled pharmacy staff may be important factors It would have been a good complement to the high level of adoption of blood sugar screening devices as many pharmacy clients have comorbidities of diabetes and hyperlipidemia. There is need for professional development efforts to prioritize hands-on training of community pharmacists in emerging technology-based diagnostics including cholesterol screening services as may be applicable to their practice setting. The service quality scores above suggest that pharmacy clients now expect these technology-driven services as part of their normal experience with pharmacy services.^9^

 The least adopted technologies for PHC among the community pharmacists seem to be mainly related to information communication technologies. For example the absence of digital technologies for decision support, such as the software for drug interactions in 210 (65%) of pharmacies will limit the capacity of the community pharmacist to detect possible adverse drug reactions and interactions which may compromise the quality of PHC services. That 103 (32%) have no social media platforms (such as WhatsApp) with which to communicate with certain clients as close-user-groups means timely dissemination of health education may be limited to in-person sessions. These deficiencies even in the midst of social influence may be related to poor perceptions of usefulness or poor technical skills (ease of use). It may also be due to poor attitude towards shifting from analogue to digital platforms in PHC service delivery.^34,35^ Detailed interrogation of the poor adoption of these digital technologies by community pharmacists is beyond the scope of this study.

 Even though as high as 258 (80%) of the pharmacies have computerized operations, this has not resulted in commensurate increase in the documentation of patient interactions as evidenced by 124 (39%) of the community pharmacists who do not document patient care services. Even a key informant stressed that routine documentation was usually limited to inventory and financial management, and not professional services such as PHC services. This suggests poor attitude towards usage due to wrong notion of job relevance for these technologies.

 Digital technologies are increasingly shaping the future of PHC.^10^ The overall picture of their adoption by respondents can best be described as rudimentary or nascent in agreement with existing local evidence.^5,9^While technologies enhance service quality, there are several other environmental determinants that must be considered in developing a framework for quality assessment and assurance in PHC in Nigeria community pharmacy.^35^

###  Factors Affecting Management of PHC Services

 The lack of awareness of the place of community pharmacy in the PHC value chain whereby community pharmacists were not integrated with the PHC infrastructure proved to be the most significant factor affecting how community pharmacists managed PHC services. This finding agrees with evidence from Iranian studies.^36,37^ However, these trends are in sharp contrast with the system in Brazil in which the Family Health Programme incorporates community pharmacists alongside other healthcare professionals as community health workers supporting the core family health teams of general practitioners and nurses in a unique blend of facility- and community-based care with impressive outcomes.^38^ Recent evidence from South Africa also shows a shift to a multidisciplinary team approach.^33,34^ In Nigeria however, the study found a lack of team approach to patient care with most community pharmacies operating as ‘silos.’ About 80% (258) of the pharmacists either agreed or strongly agreed that this was a significant challenge, as corroborated by an Australian study.^39^

 Closely related to the limitations of the “silos” effect is the capacity constraints either in terms of financial and human resources (87% of respondents agree or strongly agree), or infrastructural and technological challenges (86% of respondents agree or strongly agree). These findings agree with previous Nigerian studies.^14,15^ They also reflect aspects of a Brazilian study particularly in terms of human resources and technological challenges.^22^However, the contribution of technologies to PHC must be balanced with the need to preserve the professional identity and patient safety embedded in person-to-person interaction ofpharmacists and clients. Technologies become a threat when they take over certain key aspects of patient care from pharmacists, as highlighted by an Italian study on the emergence of online pharmacies.^40^

 It is significant to note that while insufficient time (mean score 3.83) and floor space (3.73) or uncertainties about the profitability of non-drug related services (3.82) remain challenges to management of PHC services, they were ranked among the least of the worries of the pharmacists. This seems to suggest a certain willingness to serve, driven by high personal motivations despite obvious environmental constraints.^41^This willingness to serve is signposted by the fact that Nigeria community pharmacists currently participate, pro bono, in a donor-funded anti-retroviral therapy programme with excellent outcomes in medication refill, adherence to regimen and patient retention.^42^

 The most prominent facilitator to management of PHC services was found to be confidence of the pharmacists in their managerial skills (4.35) to which 89% either agree or strongly agree. Relational attributes such as ability to leverage strong customer relationships (4.27) and customer trust (4.21) were highly rated by the pharmacists. This disposition confirms earlier evidence by the authors^43^ and emphasizes the potential for relationship marketing practices to boost customer perceptions of the quality of PHC services offered by community pharmacists in Nigeria.

###  Study Limitations

 Only 3 key informants were interviewed for this study. Though this represents a case study approach, the sample size cannot be said to provide data saturation. Again only the Southwestern zone of Nigeria was covered by the study thereby reducing the quality of generalizations**. **Moreover, the study was a cross-sectional survey which presents only a “snapshot” of the situation. A longitudinal pan-Nigeria study (involving more key informants) is therefore recommended to capture important long-term variables. Further studies are also recommended to identify factors responsible for the low adoption of certain digital technologies including cholesterol screening devices by the community pharmacists.

## Conclusion

 In Nigeria, community pharmacists offered important PHC services, the most prominent being the control and treatment of endemic diseases, disease prevention, and supply of medicines, with the potentials of immunization services currently underutilized. Deploying appropriate technologies showed strong association with improved PHC service quality but their level of adoption was rudimentary. Health system governance factors related to lack of integration of community pharmacy with the PHC architecture represents the most important challenge to effective management of these PHC services provided by community pharmacists.

 These findings imply that community pharmacists are PHC service providers and their concentration in urban centres represents a major challenge to universal health coverage. Policy action is required to incentivize their movement into rural areas. Community pharmacists should embrace more of digital technologies to enhance service quality. Policy options for integrating community pharmacies into the national PHC strategy should be explored to enhance equitable access to quality PHC in Nigeria.

## Ethical issues

 The Health Research Ethic Committee (HREC) of the Institute of Public Health, Obafemi Awolowo University, Ile-Ife Nigeria, granted approval (HREC No: IPHOAU/12/1437) for the study.

## Competing interests

 Authors declare that they have no competing interests.

## Authors’ contributions

 The study is part of the Ph.D. work of MRI,supervised by KPO. MRI formulated study objectives, reviewed literature, collected/analyzed data, wrote manuscript. KPO developed study design, approved instruments, collected/analyzed data, read and approved manuscript.
